# Oropharyngeal and nasal *Staphylococcus aureus* carriage by healthy children

**DOI:** 10.1186/s12879-014-0723-9

**Published:** 2014-12-31

**Authors:** Susanna Esposito, Leonardo Terranova, Alberto Zampiero, Valentina Ierardi, Walter Peves Rios, Claudio Pelucchi, Nicola Principi

**Affiliations:** Department of Pathophysiology and Transplantation, Pediatric Highly Intensive Care Unit, Università degli Studi di Milano, Fondazione IRCCS Ca’ Granda Ospedale Maggiore Policlinico, Via Commenda 9, Milano, 20122 Italy; Department of Epidemiology, IRCCS Istituto di Ricerche Farmacologiche Mario Negri, Milan, Italy

**Keywords:** Methicillin-resistant Staphylococcus aureus, Methicillin-sensitive Staphylococcus aureus, MRSA, MSSA, Staphylococcus aureus

## Abstract

**Background:**

As healthy children are the main reservoir of respiratory pathogens and the main cause of bacterial diffusion in the community, it could be interesting to investigate the type of screening that should be used during the early years of life in order to obtain a more precise estimate of *Staphylococcus aureus* circulation. The aim of this study was to evaluate oropharyngeal and nasal *S. aureus* carriage in otherwise healthy children and adolescents aged 6–17 years.

**Methods:**

The oropharyngeal and nasal samples were collected in December 2013 from 497 healthy students attending five randomly selected schools in Milan, Italy, using an ESwab kit, and *S. aureus* was identified using the RIDA®GENE methicillin-resistant *S. aureus* (MRSA) system.

**Results:**

Two hundred and sixty-four subjects (53.1%) were identified as *S. aureus* carriers: 129 (25.9%) oropharyngeal carriers and 195 (39.2%) nasal carriers, of whom 60 (12.1%) were both oropharyngeal and nasal carriers. Oropharyngeal carriage increased with age (p < 0.001), whereas nasal carriage decreased. There was little or no agreement between oropharyngeal and nasal carriage in any of the age groups. MRSA was identified in only three cases (0.6%), always in nasal samples. There were no differences between the carriers and non-carriers in terms of the distribution of age, gender, ethnicity, the number of siblings in the household, exposure to passive smoking, previous clinical history, allergic sensitisation, or previous influenza, pneumococcal and meningococcal vaccinations. The frequency of male children was higher among the subjects with positive nasal and oropharyngeal swabs (66.7%) than among those with positive oropharyngeal swabs alone (46.4%; p = 0.02).

**Conclusions:**

The oropharyngeal carriage of mainly methicillin-sensitive *S. aureus* is frequent in otherwise healthy children, including a relatively high proportion of those without nasal colonisation. These findings highlight the importance of adding throat to nasal screening when monitoring the circulation of *S. aureus* in the community.

## Background

*Staphylococcus aureus* carriers are at increased risk of developing *S. aureus* infections after invasive medical or surgical procedures, and more frequently develop *S. aureus* bacteremia when admitted to hospital [1]. Consequently, routine admission screening is strongly recommended in high-risk units, and the general screening of all hospitalised patients is debated [2]. The emergence of methicillin-resistant *S. aureus* (MRSA), and evidence that difficult to treat *S. aureus*-related diseases can also occur in the community has increased the importance of general screening in order to monitor *S. aureus* circulation and its susceptibility to antibiotics [3], and also explains why *S. aureus* carriage in the community has recently been periodically evaluated [4],[5].

*S. aureus* screening is usually carried out using nasal swabs because the anterior nares are considered the primary site of *S. aureus* colonisation, which can be found in the nares of up to 40% of healthy subjects [6]. Additional screening is currently considered unnecessary [1], but the findings of recent studies indicate that the throat can also be an important site of *S. aureus* colonisation, and that a relatively large number of subjects may be colonised exclusively in the throat [7]-[11], and would be missed by nasal screening. This has led some authors to conclude that *S. aureus* screening should include both nose and throat swabs in order to allow a complete evaluation [12]. Most studies of throat carriage have involved hospitalised adults, and there are very few data concerning children. However, as healthy children are the main reservoir of respiratory pathogens and the main cause of bacterial diffusion in the community [1], it could be interesting to investigate the type of screening that should be used during the early years of life in order to obtain a more precise estimate of *S. aureus* circulation.

The aim of this study was to evaluate oropharyngeal and nasal *S. aureus* carriage in otherwise healthy children and adolescents aged 6–17 years.

## Methods

### Swab collection

Oropharyngeal and nasal swabs were collected during the second and third week of December 2013 from children and adolescents attending five schools in Milan, Italy (two primary schools, two middle schools, and one high school), randomly selected with a computer-based list from the public schools considered representative of the middle class population living in Milan. Participation was completely voluntary, but was solicited by means of a letter and brochure distributed during lessons that described the characteristics of the study in detail and requested the written informed consent of both parents and the signed assent of the study subjects. Moreover, in order to maximise participation, all of the teachers reinforced the message in the week preceding the swabbing by giving detailed explanations concerning *S. aureus* carriage and its related diseases.

The study was approved by the Ethics Committees of the participating schools and the Fondazione IRCCS Ca’ Granda Ospedale Maggiore Policlinico, Milan, Italy. The children were enrolled after parental consent and subject assent had been obtained, and a brief demographic and clinical questionnaire had been completed. Children with a known, chronic underlying disease and those who had been treated with antibiotics in the previous three weeks were excluded from the study.

The swabbing was carried out in the medical room of each school at the end of the lessons on two consecutive days by a group of specifically trained pediatric residents supervised by a pediatrician (NP) using an ESwab kit containing a polypropylene screw-cap tube with an internal conical shape filled with 1 mL liquid Amies medium (cat. Number 480CE, Brescia, Copan, Italy). The oropharyngeal sampling involved pressing the tongue downward to the floor of the mouth by means of a spatula, and swabbing both tonsillar arches and the posterior nasopharynx without touching the sides of the mouth; the nasal sampling involved inserting the swab tip into one nostril and rotating it for three seconds. All the swabs were immediately transported to a central laboratory and processed within two hours.

### Identification of *S. aureus*

*S. aureus* was identified using the RIDAGENE methicillin-resistant *S. aureus* (MRSA) system (R-Biopharm AG, Darmstadt, Germany) , a multiplex real-time PCR for the direct, qualitative detection of MRSA and its differentiation from methicillin-sensitive *S. aureus* (MSSA). It has been validated for use with human nasal specimens and cultures and has a detection limit of ≤5 DNA copies per reaction [13].

DNA was extracted from the nasal and oral specimens using 200 μL of lysis buffer (provided with the kit) mixed with 100 μL of Copan swab transport media. The preparation tubes were vortexed for 60 seconds, and incubated in a heating block at 95°C for 10 minutes. The samples were then centrifuged for one minute at 12,000 rpm and supernatant was collected. After DNA isolation, the *mecA/mecC* gene, the *SCCmec/orfX junction* (type I, II, III, IV, V, VI, VII, IX and XI), and the *orfX* gene specific for MRSA were amplified by means of TaqMan technology-based assays in accordance with the manufacturer’s instructions. Each assay contains internal control DNA, which is added to the samples in the extraction step in order to detect possible PCR inhibition or DNA extraction failure. The samples were classified as negative if there was no amplification signal but the internal control DNA was positive; MRSA if they were positive for the *mecA/mecC*, the *SCCmec/orfX junction* and the *orfX* gene; MSSA if they were positive for both the *SCCmec/orfX junction* and the *orfX* gene, or only for the *orfX* gene.

### Statistical analysis

The groups were compared using contingency table analysis with the chi-squared or Fisher’s exact test, as appropriate. A Cochran-Armitage test for trend was used to compare the ordered categorical data between groups. Subgroup analyses were made on the basis of age (<10, 10–14, and ≥15 years). Agreement between the oropharyngeal and nasal swab results was examined by computing weighted kappa coefficients, using Fleiss-Cohen weights. All of the tests were two-sided, and a p value of <0.05 was considered statistically significant. The data were analysed using SAS version 9.2 statistical software (SAS Institute, Cary, NC, USA).

## Results and discussion

The parents of more than 70 % of the children attending the selected schools agreed to allow their children to take part in the study, but 35 (6.6%) of the 532 potential participants were excluded because of mild upper respiratory tract infections. Of the 497 remaining children, 264 (53.1%) were identified as *S. aureus* carriers: 129 (25.9%) oropharyngeal carriers and 195 (39.2%) nasal carriers, of whom 60 (12.1%) were both oropharyngeal and nasal carriers. This indicates that the use of oropharyngeal swabs increased the identification of *S. aureus* carriage by about 13.8%. Table 1 shows the oropharyngeal and/or nasal carriage of *S. aureus* in the study population by age group and methicillin susceptibility. Oropharyngeal carriage increased with age (p < 0.001), whereas nasal carriage decreased. However, the differences between the age groups were statistically significant only in the case of oropharyngeal carriage. There was little or no agreement between oropharyngeal and nasal carriage in any of the age groups. MRSA was identified in only three cases (0.6%), always in nasal samples.

**Table 1:** **Oropharyngeal (OP) and nasal (NAS) carriage of** ***Staphylococcus aureus*** **by healthy children living in Milan by age and** ***S. aureus*** **susceptibility to methicillin**

	Age group											
	<10 years			10-14 years			≥15 years			Total		
	n = 199			n = 213			n = 85			n = 497		
	OP	NAS	Both	OP	NAS	Both	OP	NAS	Both	OP	NAS	Both
	N (%)	N (%)	N (%)	N (%)	N (%)	N (%)	N (%)	N (%)	N (%)	N (%)	N (%)	N (%)
**MRSA**	0	1 (0.5)		0	1 (0.5)		0	1 (1.2)		0	3 (0.6)	
**MSSA**	42 (21.1)	77 (38.7)		51 (23.9)	89 (41.8)		36 (42.3)	26 (30.6)		129 (26.0)	192 (38.6)	
**Total** ***S. aureus***	42 (21.1)	78 (39.2)	21 (10.5)	51 (23.9)	90 (42.2)	24 (11.3)	36 (42.3)	27 (31.8)	15 (17.6)	129 (26.0)	195 (39.2)	60 (12.1)

MRSA: methicillin-resistant *S. aureus*; MSSA: methicillin-susceptible *S. aureus*; NAS: nasal; OP: oropharyngeal.

All of the Cohen’s kappa coefficients for agreement between total nasal and oropharyngeal *S. aureus* carriage were <0.20 (no or little agreement). Total oropharyngeal *S. aureus* (and MSSA) carriage differed by age group (p < 0.001). There was no other significant age-related difference.

Table 2 shows the general characteristics of the study population by *S. aureus* carrier status. There were no differences between the carriers and non-carriers in terms of the distribution of age, gender, ethnicity, the number of siblings in the household, exposure to passive smoking, previous clinical history, allergic sensitisation, or previous influenza, pneumococcal and meningococcal vaccinations.

**Table 2:** **Main characteristics of 497 healthy children by** ***Staphylococcus aureus*** **carriage (positive nasal or oropharyngeal swab, or both**

	All children (n = 497)	Positive (n = 264)	Negative (n = 233)	p-value
**Age (years)**				
<10	199 (40.0)	99 (49.7)	100 (50.3)	
10-14	213 (42.9)	117 (54.9)	96 (45.1)	
≥15	85 (17.1)	48 (56.5)	37 (43.5)	0.46
**Sex**				
Male	281 (56.5)	151 (53.7)	130 (46.3)	
Female	216 (43.5)	113 (52.3)	103 (47.7)	0.75
**Ethnicity** ^**a**^				
Caucasian	420 (84.8)	219 (52.1)	201 (47.9)	
Non-caucasian	75 (15.2)	45 (60.0)	30 (40.0)	0.21
**No. of siblings** ^**a**^				
0	127 (26.0)	66 (52.0)	61 (48.0)	
1	270 (55.3)	143 (53.0)	127 (47.0)	
2	69 (14.1)	38 (55.1)	31 (44.9)	
≥3	22 (4.5)	14 (63.6)	8 (36.4)	0.37
**Parental smoking habit** ^**a**^				
Both non-smokers	390 (78.5)	207 (53.1)	183 (46.9)	
At least one smoker	107 (21.5)	57 (53.3)	50 (46.7)	0.97
**Gestational age (weeks)** ^**a**^				
<37	50 (14.2)	22 (44.0)	28 (56.0)	
≥37	301 (85.8)	161 (53.5)	140 (46.5)	0.21
**Birth weight (g)** ^**a**^				
<2,500	28 (7.8)	11 (39.3)	17 (60.7)	
≥2,500	332 (92.2)	174 (52.4)	158 (47.6)	0.18
**Exclusive breastfeeding** ^**a**^				
No	113 (29.4)	54 (47.8)	59 (52.2)	
Yes	271 (70.6)	141 (52.0)	130 (48.0)	0.45
**Hospitalized for acute diseases in the last 3 months**				
No	474 (95.4)	254 (53.6)	220 (46.4)	
Yes	23 (4.6)	10 (43.5)	13 (56.5)	0.34
**Allergies** ^**a**^				
No	317 (75.3)	166 (52.4)	151 (47.6)	
Yes	104 (24.7)	55 (52.9)	49 (47.1)	0.93
**Allergic sensitization** ^**a**^				
No	338 (81.4)	172 (50.9)	166 (49.1)	
Yes	77 (18.6)	45 (58.4)	32 (41.6)	0.23
**Flu vaccination during current season** ^**a**^				
No	365 (93.8)	185 (50.7)	180 (49.3)	
Yes	24 (6.2)	11 (45.8)	13 (54.2)	0.65
**Pneumococcal vaccination** ^**a**^				
No	351 (71.0)	195 (55.6)	156 (44.4)	
Yes	143 (29.0)	67 (46.8)	76 (53.1)	0.08
**Meningococcal vaccination**				
No	303 (61.0)	168 (55.4)	135 (44.6)	
Yes	194 (39.0)	96 (49.5)	98 (50.5)	0.19

^a^The sum does not add up to the total because of missing values.

Table 3 shows the main characteristics of the children carrying *S. aureus* by site of positivity. The children with positive nasal swabs alone were younger than those with positive oropharyngeal swabs alone (p < 0.001) or positive nasal and oropharyngeal swabs (p = 0.01). The frequency of male children was higher among the subjects with positive oropharyngeal and nasal swabs (66.7%) than among those with positive oropharyngeal swabs alone (46.4%; p = 0.02). There were no statistically significant differences in any of the other considered characteristics.

**Table 3:** **Main characteristics of 497 healthy children by** ***Staphylococcus aureus*** **carriage as assessed by means of oropharyngeal (OP) or nasal (NAS) swabs or both**

	OP positive only (n = 69)	NAS positive only (n = 135)	OP and NAS positive (n = 60)
**Age (years)** ^**b**^			
<10	21 (30.4)	57 (42.2)	21 (35.0)
10-14	27 (39.1)	66 (48.9)	24 (40.0)
≥15	21 (30.4)	12 (8.9)	15 (25.0)
**Sex** ^**b**^			
Male	32 (46.4)	79 (58.5)	40 (66.7)
Female	37 (53.6)	56 (41.5)	20 (33.3)
**Ethnicity** ^**a**^			
Caucasian	61 (88.4)	109 (80.7)	49 (81.7)
Non-caucasian	8 (11.6)	26 (19.3)	11 (18.3)
**No. of siblings** ^**a**^			
0	15 (22.1)	34 (25.2)	17 (29.3)
1	42 (61.8)	71 (52.6)	30 (51.7)
2	9 (13.2)	21 (15.6)	8 (13.8)
≥3	2 (2.9)	9 (6.7)	3 (5.2)
**Parental smoking habit** ^**a**^			
Both non-smokers	55 (79.7)	108 (80.0)	44 (73.3)
At least one smoker	14 (20.3)	27 (20.0)	16 (26.7)
**Gestational age (weeks)** ^**a**^			
<37	2 (5.3)	16 (14.3)	4 (12.1)
≥37	36 (94.7)	96 (85.7)	29 (87.9)
**Birth weight (g)** ^**a**^			
<2,500	2 (5.4)	7 (6.1)	2 (5.9)
≥2,500	35 (94.6)	107 (93.9)	32 (94.1)
**Exclusive breastfeeding** ^**a**^			
No	12 (30.0)	33 (28.0)	9 (24.3)
Yes	28 (70.0)	85 (72.0)	28 (75.7)
**Hospitalized for acute diseases in the last 3 months**			
No	65 (94.2)	131 (97.0)	58 (96.7)
Yes	4 (5.8)	4 (3.0)	2 (3.3)
**Allergies** ^**a**^			
No	38 (74.5)	97 (77.6)	31 (68.9)
Yes	13 (25.5)	28 (22.4)	14 (31.1)
**Allergic sensitization** ^**a**^			
No	38 (76.0)	101 (80.8)	33 (78.6)
Yes	12 (24.0)	24 (19.2)	9 (21.4)
**Flu vaccination during current season** ^**a**^			
No	36 (90.0)	115 (95.8)	34 (94.4)
Yes	4 (10.0)	5 (4.2)	2 (5.6)
**Pneumococcal vaccination** ^**a**^			
No	54 (79.4)	94 (69.6)	47 (79.7)
Yes	14 (20.6)	41 (30.4)	12 (20.3)
**Meningococcal vaccination**			
No	45 (65.2)	83 (61.5)	40 (66.7)
Yes	24 (34.8)	52 (38.5)	20 (33.3)

^a^The sum does not add up to the total because of missing values.

^b^The frequency distribution was significantly different according to age between NAS positive and OP positive children (p < 0.001) and between NAS positive and OP and NAS positive children (p = 0.01), and according to sex between OP positive and OP and NAS positive children (p = 0.02). No other statistically significant differences emerged for any of the other characteristics considered.

This first study of oropharyngeal and nasal *S. aureus* colonisation rates in healthy school children shows that, like adults with a history of exposure to the healthcare system [7]-[11], they frequently carry *S. aureus* in the throat and that this may occur even in the absence of nasal colonisation in a relatively high proportion of cases. Although oropharyngeal sampling led to the detection of *S. aureus* carriage in fewer children than nasal sampling, it did identify about 14% of children who would have been considered negative on the basis of a nasal swab alone. The global carriage rate of more than 53% was similar to that observed in adults who have provided both nasal and oropharyngeal samples [7]-[11], but significantly higher than that reported in healthy children who have undergone nasal evaluation alone [1], thus highlighting the importance of throat sampling. In this study *S. aureus* was identified by PCR assay and not by culture. Consequently, genotyping or phenotyping data of the pathogen cannot be presented and it was not possible to establish whether more than one strain type of S. aureus could be identified in children carrying this pathogen. However, molecular techniques used in this study can be adequate for an easier and faster epidemiological evaluation of *S. aureus* carriage in the general pediatric population in order to have useful information concerning the circulation of MRSA in the community and the risk of the occurrence of community acquired (CA)-*S. aureus* illnesses that are potentially difficult to treat. The very limited circulation of MRSA found in this study could be, at least in part, due to the enrolment of children who did not receive any antibiotic treatment in the previous three weeks and for whom no antibiotic selection of oropharyngeal flora favouring MRSA emergence occurred. However, the finding suggests a marginal risk of CA disease due to MRSA in Milan: however, it is worth remembering that, although such diseases are relatively uncommon among the general population of Europe, they are considerably more frequent in intensive care units and hospital wards caring for patients with chronic diseases [14]-[16].

The fact that the rates of exclusively oropharyngeal carriage were significantly higher among the older children suggests that age may play a role in conditioning the site of colonisation. The carriage of respiratory bacteria is strictly age dependent as it is significantly higher in younger children than in adolescents [17], and is modified by vaccine use [18]. Moreover, there are data showing that the primary site of colonisation of bacteria such as *Streptococcus pneumoniae* can vary with age [19]. As it has been demonstrated that significant interactions can occur between bacterial species and between bacteria and viruses, and that these can affect the carriage rates of a given infectious agent [20], it can be hypothesised that the age-related differences in pharyngeal *S. aureus* colonisation rates may be due to time-related variations in bacterial balance in different body sites.

Regardless of age, a minority of children showed both oropharyngeal and nasal colonisation. This is different from the findings of some adult studies indicating that bacterial colonisation in the nose is almost always accompanied by colonisation in the throat [7]. However, the difference may be due to the different characteristics of the studied subjects: we only enrolled otherwise healthy children with little or no exposure to the healthcare system, whereas the adult studies mainly involved hospitalised patients. Furthermore, the adult studies also showed that dual colonisation is significantly more frequent in subjects in close contact with other *S. aureus* carriers, such as frequently hospitalised patients [9].

## Conclusions

Although it has not been established what risk oropharyngeal carriers represent in terms of bacterial spread and infection, the findings of this study highlight the importance of adding throat to nasal screening when monitoring the circulation of *S. aureus* in the community. The additional cost of throat screening could be minimised by pooling samples in the laboratory and, as in this study, using molecular methods that are more cost-effective for screening *S. aureus* than traditional cultures because of the high cost of unnecessary isolation [21].

## Abbreviations

CA: Community acquired
MRSA: Methicillin-resistant *S. aureus*
MSSA: Methicillin-sensitive *S. aureus*
NAS: Nasal
OP: Oropharyngeal
